# Interplay between Hepatitis D Virus and the Interferon Response

**DOI:** 10.3390/v12111334

**Published:** 2020-11-20

**Authors:** Zhenfeng Zhang, Stephan Urban

**Affiliations:** 1 Department of Infectious Diseases, Molecular Virology, University Hospital Heidelberg, 69120 Heidelberg, Germany; Zhenfeng.Zhang@med.uni-heidelberg.de; 2 German Centre for Infection Research (DZIF), Partner Site Heidelberg, 69120 Heidelberg, Germany

**Keywords:** hepatitis D virus, hepatitis B virus, persistence, de novo infection, cell-division-mediated spread, pattern recognition receptors, interferon response, countermeasures, Hepcludex, Myrcludex B

## Abstract

Chronic hepatitis D (CHD) is the most severe form of viral hepatitis, with rapid progression of liver-related diseases and high rates of development of hepatocellular carcinoma. The causative agent, hepatitis D virus (HDV), contains a small (approximately 1.7 kb) highly self-pairing single-strand circular RNA genome that assembles with the HDV antigen to form a ribonucleoprotein (RNP) complex. HDV depends on hepatitis B virus (HBV) envelope proteins for envelopment and de novo hepatocyte entry; however, its intracellular RNA replication is autonomous. In addition, HDV can amplify HBV independently through cell division. Cellular innate immune responses, mainly interferon (IFN) response, are crucial for controlling invading viruses, while viruses counteract these responses to favor their propagation. In contrast to HBV, HDV activates profound IFN response through the melanoma differentiation antigen 5 (MDA5) pathway. This cellular response efficiently suppresses cell-division-mediated HDV spread and, to some extent, early stages of HDV de novo infection, but only marginally impairs RNA replication in resting hepatocytes. In this review, we summarize the current knowledge on HDV structure, replication, and persistence and subsequently focus on the interplay between HDV and IFN response, including IFN activation, sensing, antiviral effects, and viral countermeasures. Finally, we discuss crosstalk with HBV.

## 1. Introduction

Chronic hepatitis D (CHD) is a global health burden manifesting as the most severe form of viral hepatitis with the accelerated development of liver fibrosis, cirrhosis, and hepatocellular carcinoma (HCC). CHD establishes itself through either simultaneous infection of hepatitis D virus (HDV) and hepatitis B virus (HBV), or through HDV superinfection in chronic hepatitis B (CHB) patients. Simultaneous infection leads to CHD in <5% of the patients [1]. In contrast, chronic infection ensues in up to 90% of patients following superinfection [2,3]. According to a recent meta-analysis, the estimated number of HDV-infected individuals is around 12 million worldwide [4]. However, due to the large gaps in reliable epidemiologic data and a diverse geographic distribution, this number might be underestimated. Accordingly, the numbers estimated by other analyses total 50 to 72 million cases worldwide [5,6].

HDV is the smallest known human virus with single-strand circular RNA genome. Numerous HDV isolates around the world have been sequenced and classified into eight genotypes (clades), HDV 1 to 8 [7,8]. Despite differing by up to 40% in sequence similarity, the genomes form similar unbranched rod-like RNA structures [6]. HDV recruits the envelope proteins of HBV for particle assembly, release, and de novo entry. Entry proceeds using the common receptor sodium taurocholate co-transporting polypeptide (NTCP) for both viruses [9,10]. Post-entry, the intra-nuclear HDV RNA replication via double rolling cycle mechanism does not require HBV-encoded polymerase but host RNA polymerases [11,12,13]. Long-term HBV-independent HDV survival was observed in a HDV mono-infected humanized mouse model [14] and also in patients after liver transplantation and the elimination of HBV [15,16,17,18]. Importantly, HDV was recently proven to be amplified through hepatocyte proliferation [19,20]. This cell-division-mediated amplification/spread might be clinically important for HDV persistence and could restrict the antiviral potential of drugs that address de novo infection (e.g., the viral entry inhibitor Hepcludex or the HDV envelopment inhibitor Lonafarnib) [21,22,23]. However, to disseminate within the human population, HDV requires HBV envelope proteins encoded by either co-infected HBV or HBV integrates expressing HBsAg.

Upon infection, host pattern recognition receptors (PRRs) sense viral genomes or replication intermediates (e.g., double-strand RNA (dsRNA)) and consequently induce innate immune responses that directly inhibit virus replication or protect the uninfected cells against subsequent infection [24]. On the other hand, viruses evolved diverse strategies to counteract these antiviral responses [25]. The interplay between viruses and the innate immune system is decisive for the outcome of infections, e.g., persistence, or clearance. However, for many years, investigations on this subject for HDV were hampered by the lack of efficient infection systems. The discovery of the viral receptor NTCP in 2012 and the development of advanced in vitro and in vivo infection models [9,10] opened the door to study not only new aspects of HDV’s replication and persistence but also molecular details of its interplay with the IFN response, which serve as the main topics of this review.

## 2. HDV Structure, Replication, and Persistence

### 2.1. HBV/HDV Virions and HDV RNAs

**Figure 1:**
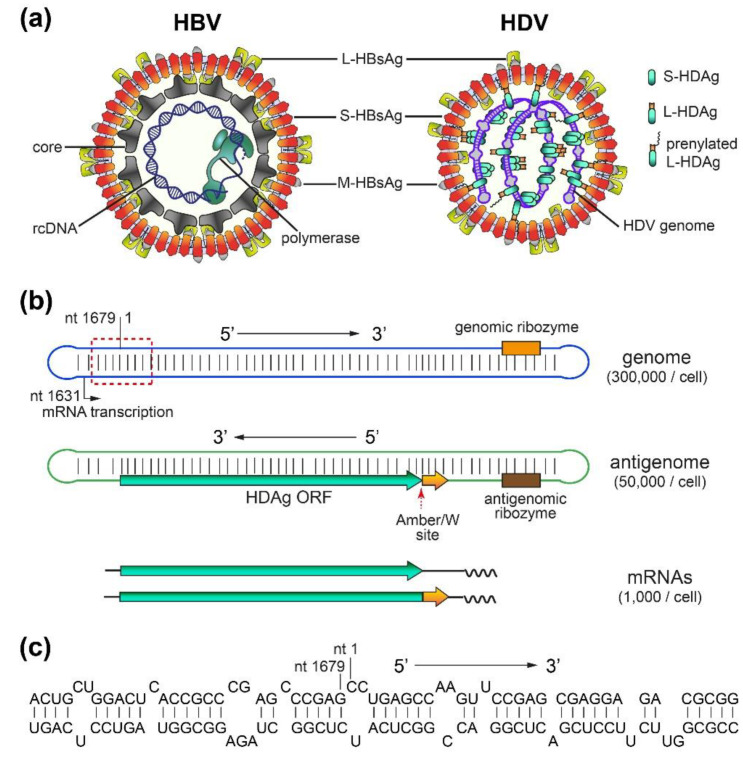
Hepatitis D virus (HDV) and Hepatitis B virus (HBV) virions, and HDV RNAs. (**a**) Schematic representation of HDV and HBV virions. Both viruses share the same envelope containing three HBV envelope proteins: large- (L-), medium- (M-), and small- (S-) HBsAg. HDV (right panel) has a ribonucleoprotein (RNP) complex inside. The RNP consists of the HDV genome and two isoforms of hepatitis D antigen (HDAg), L- and S-HDAg. A portion of L-HDAg is prenylated, which is needed for its association with S-HBsAg [35]. On the other hand, HBV (left panel) has a nucleocapsid inside the envelope. The nucleocapsid consists of an HBV core protein shell and relaxed circular HBV DNA (rcDNA), with the latter associated with HBV polymerase. (**b**) HDV genome, antigenome and mRNAs. The HDV genome is a single-strand, negative-sense, circular RNA. It forms an unbranched rod-like structure due to its high degree of intramolecular base-pairing. The HDV antigenome is complementary to the genome and is predicted to form a similar structure to the genome. Two mRNAs encoding either S-HDAg or L-HDAg are transcribed using the genome as a template. Ribozymes, the ADAR1 editing (Amber/W) site, mRNA transcription starting site, and HDAg open reading frame (ORF) are indicated. Arrows indicate the 5′ to 3′ direction. (**c**) Structure of a representative region of the genome (red dash line box in (**b**)) consisting of short stems and bulges.

The HDV virion, approximately 36 nm in diameter, consists of a ribonucleoprotein (RNP) core complex and an HBV-encoded envelope (Figure 1a). The RNP complex contains an approximately 1.7 kb, single-stranded, circular, negative-sense RNA genome and two isoforms of the HDAg—small (S-HDAg) and large (L-HDAg). The envelope is composed of an endoplasmic reticulum (ER)-derived lipid bilayer embedding the three HBV envelope proteins: small (S-), medium (M-), and large (L-) HBsAg.

HDV replication produces three species of RNA: genomic, antigenomic, and mRNA (Figure 1b). Due to its high degree of intramolecular base pairing (approximately 74%), the HDV genome folds into rod-like structural elements [26,27] that consist of short dsRNA stems interrupted by single-stranded bulges, as depicted in Figure 1c. Analyses of 41 HDV isolates indicated that the longest consecutive base-paired regions range from 9 to 15 bp [28]. At a higher-order level, the HDV genome associates with S-HDAg and forms an RNP, which is proposed to be organized in a nucleosome-like structure during replication [29]. Antigenome and mRNAs are synthesized using the genome as a template. As a complement of the genome, the antigenome is predicted to fold into similar secondary structure elements to the genome. In contrast, the HDV mRNAs, lacking the elements of the complementary strand, likely do not form similar structures to genomic/antigenomic RNAs.

Despite sharing the same envelope, HBV and HDV differ in their virion architecture and size: HBV is larger (around 42 nm) and contains an icosahedral nucleocapsid within the envelope (Figure 1a). This nucleocapsid has been shown to organize the envelope by specifically interacting with the matrix domain in the L surface protein [30]. HDV presumably lacks such an envelope organization since its RNP complex is less structured and the interaction of HDAg with HBsAg depends only on a hydrophobic prenylation residue within the C-terminus of L-HDAg and the cytosolic loop in the S-domain. Consequently, the stoichiometry of envelope proteins can vary in HD virions [31]. Notably, HDV RNPs can also be enveloped as a non-infectious particle containing only the S surface protein [32]. Due to their intrinsic self-assembly competence, HBV envelope proteins also form empty subviral particles as spheres and filaments. These particles far exceed the number of virions and make up most of the HBsAg, which is used as a diagnostic marker [33,34].

### 2.2. HDV Replication Cycle

**Figure 2:**
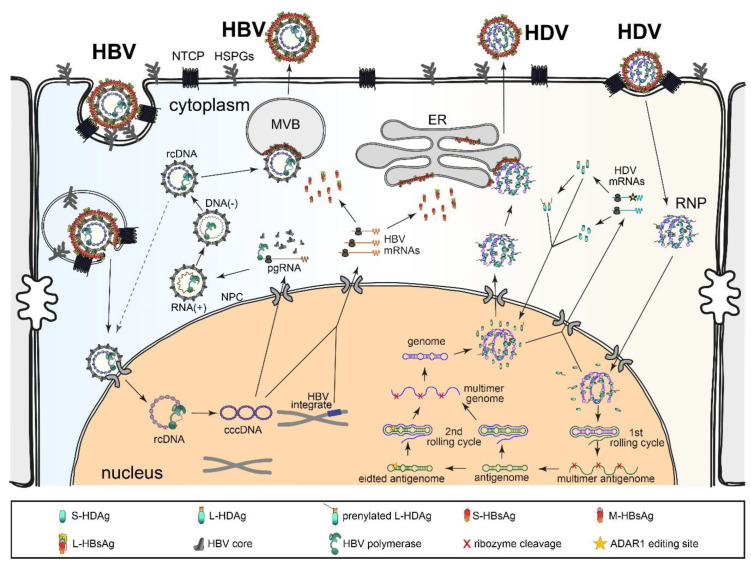
HDV and HBV life cycles. Right half: HDV life cycle. HDV virions first attach to heparan sulfate proteoglycans (HSPGs) [36,37,38,39] and then to the viral receptor sodium tautocholate co-transporting peptide (NTCP) [9,10]. After membrane fusion, the ribonucleoprotein (RNP) is released into the cytoplasm and further transported to the nucleus where RNA replication occurs [43,44]. The genome serves as the template for the first rolling circle amplification. The resulting antigenome multimers are cleaved in cis by the intrinsic ribozyme and ligated into circular antigenome monomers [35,45]. After a second rolling cycle using the antigenome as the template, HDV genome multimers are synthesized and self-cleaved to produce circular HDV genome monomers. The HDV antigenome might be edited by cellular adenosine deaminases acting on RNA 1 (ADAR1), yielding an extended HDAg ORF that produces L-HDAg [46]. These genomes, with or without ADAR1 editing, are used as the template for mRNA transcription. The mRNAs are translated into S-HDAg and L-HDAg. A portion of the L-HDAg molecules are prenylated for envelope acquirement [35]. S-HDAg and L-HDAg are transported into the nucleus to regulate virus replication or bind to the genome to form RNP, which is exported to the cytoplasm. Through the interaction between L-HDAg and S-HBsAg, RNP acquires an envelope and is released through the endoplasmic reticulum (ER)–Golgi secretory pathway. Left half: HBV life cycle. After binding to HSPG and NTCP, HBV is internalized through endocytosis [47]. The fusion of the HBV envelope with the endosome membrane releases the nucleocapsid, which is further transported to the nuclear pore complex (NPC) where rcDNA is imported into the nucleus. The rcDNA is processed into covalently closed circular DNA (cccDNA). This cccDNA serves as the template for HBV mRNAs and pregenomic RNA (pgRNA), with the latter captured in the HBV capsid and reverse-transcribed to the DNA of the progeny virus via HBV polymerase. The progeny HBV is considered to be secreted through a multivesicular body (MVB) [48]. Notably, HBV DNA might be integrated into cellular chromosomes [49,50]. These integrates can produce HBV envelope proteins that support HDV packaging [50,51,52,53].

The replication cycle of HDV is depicted in Figure 2 (right half). To initiate infection, HDV virions associate with heparan sulfate proteoglycans (HSPGs), e.g., glypican-5, on the surfaces of the hepatocytes [36,37,38,39]. Attachment to HSPGs is required but insufficient to mediate productive infection. However, attachment promotes subsequent and highly specific interaction with the receptor NTCP at the basolateral membranes of the hepatocytes [9,10]. The myristoylated N-terminal 75 amino acids of the PreS1 domain of the L surface protein are responsible for this interaction [40]. Besides NTCP, the epidermal growth factor receptor (EGFR) has been recently described as a co-factor for HBV/HDV entry by regulating endocytosis and sorting incoming viral particles [41,42]. The internalization and membrane fusion steps of HDV entry are proposed to be similar to those of HBV (see below). However, direct comparative studies of both viruses during these stages are rare.

After the release of the HDV RNP into the cytoplasm of hepatocytes, the subsequent steps of HDV replication are HBV-independent. The HDV RNP is transported to the nucleus where RNA replication initiates [43,44]. The incoming genome serves as the template for the first round of rolling circle amplification (RCA), generating linear multimeric antigenomic RNAs that are self-cleaved by the intrinsic antigenomic ribozyme and ligated to form antigenomic circular monomers [35,45]. Similarly, genomes are produced by the second round of RCA using the newly generated antigenomes as the template and further processing through the genomic ribozyme. Unlike other negative-strand RNA viruses, HDV does not encode an RNA-dependent RNA polymerase (RdRP). Instead, it redirects cellular DNA-dependent RNA polymerases (Pols) for RNA replication. Strong evidence has demonstrated that RNA Pol-II is responsible for HDV genome and mRNA synthesis [54,55,56]. However, the Pol(s) responsible for HDV antigenome synthesis are debatable [12,55,56]. Importantly, S-HDAg is essential in these processes [57,58]. The incoming S-HDAg as part of the RNP is sufficient to initiate replication. It is unclear how HDV hijacks normally DNA-dependent Pol to an RNA template. The mainly double-stranded nature of the HDV genome/antigenome and S-HDAg are considered crucial for this template switch [59,60,61].

Over the course of replication, a fraction of the newly synthesized antigenomic RNA becomes edited by the host adenosine deaminase acting on RNA 1 (ADAR1) at the amber stop codon of the S-HDAg open reading frame (ORF), changing the UAG to UIG [46,62]. The inosine (I) is then recognized as guanosine (G) in the subsequent replication round, leading to the introduction of a tryptophan codon (UGG). Consequently, the ORF is extended by 19 or 20 amino acids (genotype-dependent), leading to its translation into L-HDAg. In contrast to S-HDAg, L-HDAg inhibits HDV RNA replication [63,64] and promotes progeny virion assembly [65,66]. For this process, a fraction of L-HDAg becomes prenylated via the cellular farnesyl transferase at the Cys residue in the C terminal CXXQ motif within the extension [35]. The de novo synthesized genomic HDV-RNA forms an RNP complex by incorporating prenylated and non-prenylated L-HDAg in addition to S-HDAg [45,67,68]. The RNP then becomes enveloped through budding into an ER-derived lipid bilayer carrying the three HBV envelope proteins encoded by either covalently closed circular DNA (cccDNA) or integrated HBV DNA (see below) [51,52]. The prenylation of L-HDAg is essential for envelopment through interaction with the cytoplasmic domain of S-HBsAg [35,69]. For a detailed description of the HDV life cycle, see the following reviews [67,70,71].

Except for the initial association with HSPGs and NTCP, the HBV life cycle completely differs from that of HDV (Figure 2 left half). HBV is considered to be uptaken in a clathrin- and dynamin-dependent manner with membrane fusion occurring after endocytosis [47]. However, other pathways, like caveolin-1 mediated endocytosis, were also shown to be important for HBV entry, which might be cell-type-specific (reviewed in [72,73]). Next, the HBV nucleocapsid is transported to the nuclear pore complex (NPC), where release of the relaxed circular DNA (rcDNA) into the nucleus leads to rcDNA repair and the formation of cccDNA. This process depends on a set of cellular enzymes like DNA polymerase δ and DNA ligase I [74]. The cccDNA serves as the template for HBV mRNAs and pregenomic RNA (pgRNA), with the latter being incorporated into the HBV capsid and reverse transcribed to the (-)-strand and subsequently (+)-strand DNA of the progeny virus via HBV-encoded polymerase. Notably, HBV replication also produces double-strand linear DNA (dslDNA), which can be integrated into cellular chromosomes [49,50]. Although these integrates are unable to produce infectious HBV because they consist of incomplete HBV DNA, they produce HBV mRNAs encoding HBsAg. This cccDNA-independent production of HBsAg is sufficient to support HDV assembly and secretion in an infected cell [50,51,52,53]. Notably, such integrated HBV DNA may also serve as an important source of HBsAg in chronically infected patients [75] and provide considerable replication space for HDV in the livers of infected patients.

### 2.3. HDV Spread and Persistence

**Figure 3:**
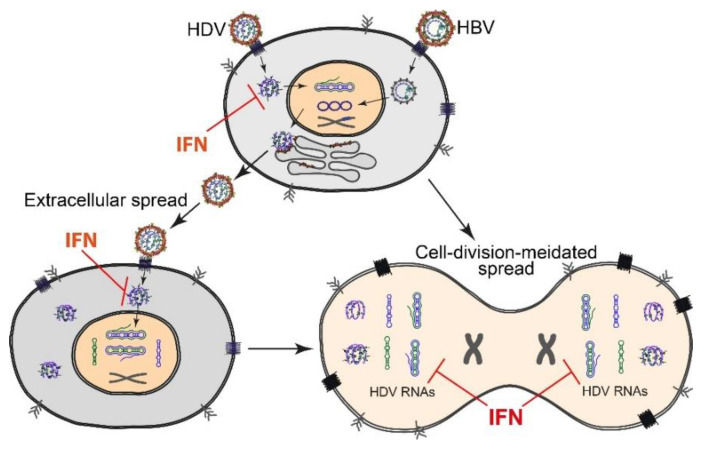
HDV spreading pathways and the targets of the interferon (IFN) response. Left: de novo infection-mediated extracellular spreading pathway. HBV/HDV co-infection produces progeny HDV that infect neighboring intact hepatocytes. The IFN response inhibits early stages of HDV de novo infection but does not significantly impair HDV RNA replication in the nucleus. Right: cell-division-mediated HDV spread. HDV survives cell division and efficiently establishes replication in both daughter cells. The IFN response causes efficient degradation of HDV RNA during cell division and/or prevents the re-establishment of replication in daughter cells.

HDV persistence relies on continuous replication and viral spread to achieve long-term maintenance of its viral RNA. HDV uses HBV envelope proteins for assembly and de novo infection, a process that is crucial for HDV spread and persistence (Figure 3). This extracellular route of HDV spread can be efficiently blocked by the entry inhibitor Hepcludex/bulevirtide (formerly Myrcludex B) but also indirectly by HDV secretion inhibitors like Lonafarnib. Hepcludex is a myristoylated oligopeptide (47-aa) derived from the preS1-domain of the HBV L-HBsAg. It efficiently blocks NTCP, the receptor of HDV/HBV, thereby inhibiting the de novo initiation of replication in vitro [76,77] and in mice transplanted with primary human hepatocytes (PHH) [22,23,78,79]. Due to its safety and efficacy in two phase II clinical trials (Myr-202 and Myr-203), Hepcludex was conditionally approved, with marketing authorization (CMA) provided by European Medicines Agency (EMA) in July 2020. Lonafarnib is an investigational drug that inhibits L-HDAg prenylation and, consequently, HBV envelope acquisition [80,81].

Although blocking extracellular HDV spread significantly suppresses HDV, as well as propagation in cell culture models, animals, and patients, accumulating evidence indicates another mode of maintenance independent of de novo cell entry: (i) HDAg-positive hepatocytes were detected after liver transplantation for >1 year in the absence of HBV DNA and serum-HBsAg [17,18], and (ii) HDV mono-infection persisted in humanized mice for at least six weeks in the absence of HBV and could be rescued by HBV superinfection [14]. An alternative HDV spreading pathway, cell-division-mediated spread (Figure 3), was reported recently in cell lines [19,20] and PHH transplanted mice [19]. In contrast to de novo infection, this pathway is HBV-independent and refractory to Hepcludex [19,82] and Lonafarnib [63]. HBV-independent HDV persistence is also supported by the discovery of HDV-like agents from rodents [83], snakes [84], birds [85], fish, amphibians, and even invertebrates [86]. None of these agents were found to be associated with an animal hepadnavirus. Moreover, bioinformatic analyses do not predict the encoding of prenylated L-HDAg-like antigens by these agents. Another study showed that HDV RNP could be packaged into the envelope proteins of vesiculo-, flavi-, and hepaciviruses in vitro, allowing the egress of HDV RNPs from cells and subsequent entry into cell lines expressing the respective receptors [87]. It is controversial whether HDV is able to use the envelopes of non-hepadnaviruses for dissemination in patients [88,89,90]. HBV envelope-independent spread may play a yet-unknown role in HDV persistence in CHD patients and challenge the effect of drugs interfering with HD virion production or de novo virus entry. Finally, the possibility of the long-term maintenance or reactivation of silenced HDV RNA at the single cell level cannot be ruled out. The latter mechanism remains an unproven hypothesis but is supported by occasional observations of non- or low-replicating RNA in cell culture systems [10,91].

## 3. IFN Signaling during RNA Virus Infection

**Figure 4:**
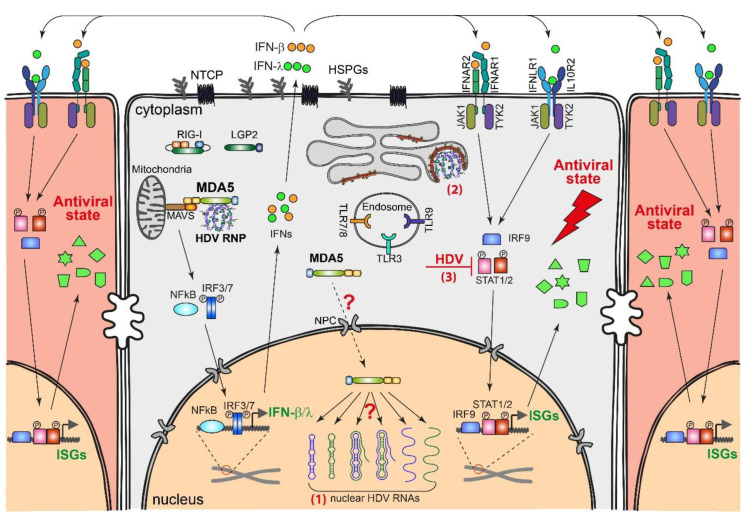
HDV-induced IFN response and possible HDV countermeasures. HDV RNA in the cytoplasm; likely, the RNP complex is recognized by the pattern recognition receptor (PRR) MDA5 [106]. This recognition activates the mitochondrial antiviral signaling protein (MAVS) on the mitochondria and downstream transcription factors, likely IFN regulatory factor (IRF) 3/7 and nuclear factor-κB (NFκB). The activated transcription factors are translocated into the nucleus and initiate the transcription of IFN-β/λ. Secreted IFN-β/λ binds to their receptors (IFNAR1/IFNAR2 for IFN-α/β and IFNLR1/IL10R2 for IFN-λ) on the infected cell or neighboring cells, which further activates Janus kinases (JAK) 1/2, tyrosine kinase (TYK) 2, and transcription factors signal transducer and activator of transcription (STAT) 1/2 and IRF9. STAT1/2 and IRF9 are translocated into the nucleus and activate hundreds of IFN-stimulated genes (ISGs), which directly inhibit HDV replication and protect the uninfected cells against subsequent infection. It is unknown whether MDA5 can also be transported to the nucleus and capture nuclear HDV replication intermediates. HDV may counteract the IFN response through different strategies: (1) HDV may replicate in a “safe” compartment, the nucleus, to avoid exposure of the replication intermediates to PRRs; (2) HDV genomic RNA in the cytoplasm may fold into RNP with HDAg and bud into an HBV envelope to avoid being recognized by the PRRs; and (3) HDV may directly inhibit STAT1/2 activation.

As the first line of defense, the innate immune system plays an essential role in the suppression of invading viruses through the activation of direct antiviral responses (e.g., the IFN response) [92] and mediating the induction of adaptive immune responses [93]. Its level of speed and strength can determine the outcome of an infection, i.e., clearance or persistence, with the latter often associated with chronic inflammation [94]. An overview of the IFN activation and signaling pathway is depicted in Figure 4. Cellular innate immune responses are initiated by recognition of pathogen-associated molecular patterns (PAMPs), e.g., viral genomes and replication intermediates like double-stranded RNA (dsRNA). The PAMPs are recognized by specific PRRs like toll-like receptors (TLRs) and retinoic acid inducible gene I (RIG-I)-like receptors (RLRs) [95,96]. This recognition triggers a cascade of signaling events that lead to the production and secretion of type I (IFN-α/β) [97,98] and type III interferons (IFN-λ) [99,100]. These IFNs amplify the signal in a paracrine and autocrine manner by binding to their cognate IFN-receptors (IFNAR1/IFNAR2 for type I IFN and IFNLR1/IL10R2 for type III IFN) on the membranes of infected and non-infected neighboring cells [101], which activates Janus kinases 1/2 (JAK1/2), tyrosine kinase 2 (TYK2), signal transducer and activator of transcription 1/2 (STAT1/2), and IFN regulatory factor 9 (IRF9) and consequently induces hundreds of IFN-stimulated genes (ISGs) to exert direct or indirect antiviral activities [102,103,104]. For more details about innate immune responses during virus replication, see the following reviews [104,105].

RLRs, including RIG-I, melanoma differentiation antigen 5 (MDA5), and laboratory of physiology and genetics 2 (LGP2), are important PRRs that sense viral RNA during infection. RIG-I and MDA5 share a high sequence similarity and the same protein domain architecture, consisting of the N-terminal tandem caspase activation and recruitment domain (CARD), the central DExD/H box motif helicase domain, and the zinc-binding C-terminal domain (CTD) [107,108]. However, they have distinct specificities towards ligands. RIG-I predominantly recognizes short 5-tri- or diphosphorylated dsRNA [109,110,111]. Sendai viruses [112,113], Influenza A virus [114,115], Dengue virus, and Zika virus [116], among others, produce RIG-I ligands during replication. MDA5 senses long double-stranded RNA and higher-ordered RNA structures [117,118]. Replication intermediates of, e.g., encephalomyocarditis virus [97,118] and hepatitis C virus (HCV) [119] are sensed by MDA5. LGP2 is the least investigated of the three RLRs. Studies demonstrated that LGP2 binds to double-stranded ends of the RNA [120,121,122]. Nevertheless, there might be other types of LGP2 ligands that remain unknown. LGP2 cannot initiate RLR signaling because of the lack of a CARD domain [108]. Several lines of evidence suggest that LGP2 augments MDA5-dependent signaling, likely by promoting the formation of MDA5-RNA complexes [123,124]. However, LGP2 inhibits RIG-I-dependent signaling, possibly via direct competition for ligand binding [125,126]. In the absence of ligands, the signaling of RIG-I is blocked by conformational changes and shielding of its CARD [127]. Upon RNA detection, both RIG-I and MDA5 change their structures and oligomerize on the RNA ligand, which further triggers oligomerization of the mitochondrial antiviral signaling protein (MAVS) through the interaction of their CARD domains [128]. The MAVS oligomer recruits downstream factors and activates a signaling cascade that leads to the expression of IFNs [129]. Notably, current knowledge regarding the specificity of ligands and the mode of interaction between PRRs and ligands is mainly based on in vitro studies using several model viruses or artificial ligands. The viral RNA structures formed during replication are considered to be highly diverse for different viruses. Therefore, the mechanisms of recognition and innate immune activation might be distinct and should be investigated separately for each virus.

## 4. IFN Response during HDV Infection

### 4.1. HDV-Induced Innate Immune Responses

With its highly back-folding RNA structure and mostly host-dependent replication strategy, investigations into HDV may provide unique insights for understanding the interplay between viruses and the innate immune system. HDV-induced innate immune responses have been reported in HDV-infected cell lines, primary human hepatocytes (PHH), and animal models [106,130,131,132,133,134]. HDV mono-infection and HBV/HDV co-infection induced strong type I IFN- and ISG-responses in differentiated HepaRG cells [130]. This was confirmed in HDV-infected PHH and NTCP over-expressing HepG2 and HepaRG cells, where HDV replication mainly induced IFN-β and IFN-λ but not IFN-α [106]. Transcriptome analysis of HDV-infected HepaG2-NTCP cells showed a set of ISGs to be among the most upregulated genes [106]. In a humanized uPA/SCID/beige (USB) mouse model repopulated with PHH, HBV/HDV co-infection activated IFNs, ISGs, and human cytokines (e.g., IP10 and TGF-β), while HBV mono-infection remained stealth [132]. HDV replication in mouse hepatocytes was demonstrated in an earlier study through hydrodynamic injection using naked HDV complementary DNA (cDNA) or RNA [135]. Following the identification of NTCP as the HBV/HDV receptor, NTCP transgenic mice partially supporting HDV replication were generated. Using these NTCP-transgenic mice, He et al. demonstrated HDV-induced type I IFN and ISG responses through an analysis of the liver transcriptome [131]. HDV-activated innate immune responses were further confirmed by Benjamin et al. [134] using similar transgenic mice and by Suarez-Amaran et al. [133] using an Adeno-associated virus (AAV) transduction-mediated mouse model.

Although the studies above have provided compelling evidence that HDV replication induces IFN response, HDV is a relatively moderate stimulator compared to some other RNA viruses, e.g., Sendai- and Mengo viruses [106] or synthetic polyviruses (I:C) [136]. Notably, most of these studies were based on acute HDV infections with relatively high levels of HDV replication. Whether this holds true in chronically infected patients remains to be investigated. In addition, the HDV viral load and intra-liver replication levels vary dramatically among patients, so the strength of HDV-induced innate immune responses also likely vary accordingly.

### 4.2. Innate Immune Sensing of HDV Replication

The sensing of PAMPs by PRRs triggers the activation of innate immune responses. In contrast to some viruses, e.g., the Influenza virus, whose incoming genomes can activate an IFN response [137]. UV-inactivated HDV failed to activate this response [106], indicating the requirement of active HDV RNA replication. The depletion of RIG-I, MDA5, and TLR3 in HepG2-NTCP and HepaRG-NTCP cells proved that MDA5 is the key sensor in recognizing HDV replication [106]. This finding is in line with a previous observation in a mouse model demonstrating the essential role of MAVS, a key downstream adaptor of MDA5, in innate immune activation during HDV replication [133].

The mechanism of HDV RNA recognition by MDA5 is still unclear. MDA5 is preferentially located, and acts, in the cytoplasm. However, HDV RNA replicates in the nucleus, and its single- and double-stranded genomic and antigenomic RNA intermediates are confined to this compartment. Accordingly, it is unlikely that these intermediates are available for sensing at the site of the primary location of MDA5. However, the progeny HDV genomes are delivered to the cytoplasm where they can be captured by MDA5. A recent study demonstrated that a minor portion of RIG-I could reside in the nucleus and capture nuclear RNP complexes of the Influenza A virus [138]. Although the contribution of this recognition in innate immunity activation is still unclear, the possibility that nuclear-localized HDV RNAs are captured by PRRs like MDA5 cannot be ruled out. Except for MDA5, the roles of other cellular factors (e.g., LGP2) and viral factors (HDAg and HBV envelope proteins) in the innate immune recognition of HDV RNA remain unknown.

Besides infected hepytocytes, innate immune cells like dendritic cells (DCs) and macrophages may also produce IFNs during HDV infection [139]. For example, plasmacytoid DCs (pDCs) can dedicate an astonishing 60% of their transcriptional activity to make type I IFN during activation [140]. Lacking the receptor NTCP, these cells are not the natural target of HDV. However, they may capture viral RNA via unspecific uptake pathways. As known from HCV, extracellular vesicles (EVs) containing viral replication intermediates can be secreted from infected cells and transferred to DCs and macrophages [141,142]. However, depending on the virus, the concentration of viral RNA containing EVs may be below the threshold for activating general cellular innate immune responses [143]. Moreover, it has been demonstrated that pDC can also capture viral RNA more efficiently by directly contacting infected cells and forming an interferogenic synapse that enables efficient EV-mediated viral RNA delivery for IFN activation [143,144,145,146]. EVs secreted from HDV-infected hepatoma cell lines and PHH were analyzed recently [147]. These EVs can activate peripheral blood mononuclear cells (PBMCs) and macrophages in vitro, leading to the production of pro-inflammatory cytokines (TNF-α, IFN-γ, IL6, etc.) [147]. However, the level of IFN activation was not reported in this study. It is unclear yet whether these EVs can deliver HDV RNA to PBMCs and macrophages to activate IFNs and, if so, which species of HDV RNA are transferred for this activation. Further investigations are, therefore, needed to understand the possible roles of innate immune cells in physiologically relevant models, e.g., co-cultures of HDV-infected hepatocytes with innate immune cells, mouse models, and—ideally—patient samples.

### 4.3. Effect of IFN Response on HDV Replication

As an off-label drug, IFN-α has been used for treating CHD patients since the 1980s. Although its rate of HDV elimination is low, IFN-α therapy decreases the HDV viral load in most patients [148,149,150,151]. A similar effect was observed in HDV-infected humanized mice upon IFN-α/λ treatment [152]. Regarding the mode of action, an early study showed that the IFN-α treatment of HDV-infected PHHs preferentially affected the early stages of infection (entry, including the establishment of replicative intermediates). Moreover, a high dose of IFN-α (600 units/mL) was needed to achieve an effect, which is 300 times greater than the dose needed for the inhibition of vesicular stomatitis virus (VSV) [153]. In a recent study, poly (I:C) was used to artificially activate the cellular IFN response either 12 h before or 12 days after HBV/HDV infection. Pretreatment significantly inhibited the replication of both viruses, while late treatment only affected HBV replication [136]. This was confirmed by our study using NTCP expressing HepG2 and HepaRG cells, where early treatment (d1–7) with IFN-α (100 IU/mL) and IFN-λ1 (10 ng/mL) reduced HDV infection by around 50%, while late treatment (d5-11) barely affected HDV replication [106]. Besides exogenous IFN treatment, we also investigated the long-term effects of the virus-induced IFN response on HDV replication in this study. Intracellular HDV RNA replication was comparable in HepaRG-NTCP regardless of MDA5 depletion within the first week post-infection. However, it was significantly decreased in cells with intact MDA5 at late time points, e.g., 7.6-fold lower compared to that in MDA5-depleted cells at day 23 post-infection [106]. Thus, a long-term virus-induced IFN response also restricts intracellular HDV RNA replication. This effect was also supported by studies using immune-competent mouse models where the depletion of innate immune responses by knocking out the IFN-α/β receptor [131] or MAVS [133] promoted HDV replication in mouse hepatocytes.

Besides de novo infection and intracellular HDV RNA replication, the effect of IFN on the newly discovered cell-division-mediated HDV spread was also evaluated recently. This spread was very efficient in the absence of an IFN response (e.g., in HuH7-NTCP cells). However, it was significantly suppressed by IFN-α/λ treatment and the HDV-induced IFN response in innate immune competent cells, e.g., HepaRG-NTCP cells [20]. In contrast to the slow and mild effects in resting cells, the suppression of HDV replication via IFN response is rapid and robust in mitotic cells. However, this seems contradictory to the overall transcription regulation during mitosis. Studies have demonstrated that IFN/ISG production is downregulated, and, consequently, antiviral activity is low during mitosis [154,155], which is likely due to the chromosome condensation and global repression of cellular transcription in the G2/M phase [156,157]. The replication of some IFN-sensitive and cytoplasmic replicating viruses like VSV-ΔM51 is also enhanced in this phase due to the reduction of cellular antiviral activity [154]. In contrast to VSV-ΔM51, HDV is a nuclear replicating virus. One possible explanation for the stronger HDV suppression by IFN responses during cell division is that the nucleus-resident HDV replication intermediates in resting cells are exposed to PRRs and ISGs during mitosis because of the destruction of the nucleus. Therefore, although the global IFN response is lower in this phase, the HDV-specific antiviral activity might be more efficient than that in HDV-infected resting cells. In addition, due to the reorganization of nuclei after cell division, HDV likely needs to “re-establish” the replication system in the daughter cells. Since the IFN response can efficiently impair the establishment of HDV replication during de novo infection (see above), it might exhibit similar inhibition in this re-establishment.

Discovery of this new mode of action by IFN also provides insights for CHD therapy. As mentioned in Section 2.3, cell-division-mediated HDV spread is refractory to drugs like Hepcludex [19,82] and Lonafarnib [82] targeting de novo infections. A combination of IFN and these inhibitors is predicted to provide better antiviral activity by targeting both HDV-spreading pathways. This hypothesis is supported by recent clinical studies demonstrating strong synergistic antiviral effects with a combination of pegylated IFN-α and Hepcludex/Lonafarnib [158,159,160,161]. To verify the effect of this combination treatment in vitro, we recently generated a model supporting both spreading pathways using HuH7-NTCP cells stably expressing HBV envelope proteins. As observed in the clinical studies, a combination of inhibitors targeting both spreading pathways (e.g., Hepcludex plus IFN-α) in this model showed strong synergism against HDV [82]. More information about novel HDV antivirals and combination therapies is reviewed elsewhere [162,163].

The effect of IFN response on HDV assembly and cellular particle egress from infected cells has not been studied so far. This is mainly due to the lack of a suitable HBV/HDV co-infection system and the rarity of “true” co-infection at the single cell level. In response, hepatoma cell lines stably expressing NTCP and the three HBV envelope proteins under endogenous promoter control have been constructed recently [52,53]. Such models mimic the possible replication of HDV in HBsAg-expressing hepatocytes after the integration of double-strand linear HBV DNA [50], as reported frequently in preferentially hepatitis B e antigen (HBeAg)-negative patients [51,75]. The efficient secretion of progeny viruses was achieved after HDV infection [52] or the stable integration of HDV-encoding cDNA [53]. IFN-α/λ treatment in these models indicates that IFN does not significantly affect HBsAg secretion and HDV production. Notably, these studies were performed on hepatoma cell lines, where HDV secretion efficiency was found to be much lower than that in the liver. Further investigations using better models are needed to clarify the effect of the IFN response on HDV particle assembly and release.

### 4.4. Countermeasures by HDV

HDV replication accumulates up to 300,000 copies of genomic RNA and 50,000 copies of antigenomic RNA per cell (Figure 1) [26]. To avoid being sensed by the host PRRs and activating a high level of innate immune responses, HDV might have evolved strategies to hide, mask, or shield these viral RNAs. Firstly, HDV uses a relatively “safe” compartment, the nucleus for RNA replication. Most of the host RNA sensors and IFN-induced effectors are localized in the cytoplasm. Therefore, HDV RNA replication intermediates are likely to be inaccessible to them. This speculation is indirectly supported by the observation that only HDV mRNA, but not the genome and antigenome, is targetable by siRNA [164]. Secondly, the progeny genomes in the cytoplasm are protected by HDAg and HBV envelope proteins. HDAg and HDV genomes form the RNP complex, which is highly compressed [165,166] and might even be resistant to nuclease [167], indicating its spatial inaccessibility to at least some cellular factors. Recognition by MDA5 may also be dampened by the assembly of RNP. Thirdly, besides escaping from PRRs, HDV may also directly counteract the IFN signaling pathways. Following the transfection of hepatoma cells with an HDV cDNA, an earlier study showed that HDV could inhibit IFN-α-induced STAT1/2 phosphorylation and nuclear translocation and, therefore, downregulate the transcription of ISGs, such as Mx1, 2′,5′-OAS, and PKR, in response to IFN-α treatment [168]. Similarly, poly (I:C)-activated IFN production was also impaired in HDV replicating 293 stable cells, and this impairment could also be achieved by expressing S-HDAg, although with less efficiency [153]. Notably, these studies were performed using either cDNA-driven HDV replication systems or the overexpression of HDAg. Using a humanized mouse model, it was later shown that a portion of human hepatocytes with a high-level HDV replication were deficient in STAT1 activation [132]. In contrast, recent work from our group demonstrated profound ISG (e.g., Mx1) induction in HDV-replicating HepG2-NTCP and HepaRG-NTCP cells [106]. Further investigations using authentic infection systems are needed to clarify these discrepancies.

## 5. Crosstalk between HDV-Induced IFN Response and HBV

### 5.1. The Role of HBV in IFN Response Activation during HBV/HDV Co-Infection

In contrast to HDV, HBV replication does not activate significant innate immune responses [132,169,170,171,172,173,174]. Although some publications described HBV-induced IFN or pro-inflammatory responses [175,176,177,178,179], these responses are usually low and temporary compared to those activated by HDV. Other studies showed that HBV pgRNA and DNA could be substrates of cellular PRRs [174,175,178,179]. However, such RNA/DNA is likely not reachable by PRRs in the cytoplasm due to shielding by the HBV capsid (Figure 2).

Regarding their effects on HDV, HBV envelope proteins can wrap the HDV RNP complex, which likely prevents the recognition of HDV RNA by PPRs. This hypothesis has not been experimentally tested so far, mainly due to the low HBV/HDV co-infection rates in most of the available models. However, the recently generated stable cell lines expressing HBV envelope proteins and supporting the full HDV life cycle mentioned above [52,53] might be good models for investigating the role of HBsAg in the innate immune sensing of HDV RNA.

### 5.2. Effect of HDV-Induced IFN Response on HBV Replication

The repression of HBV is frequently observed in CHD patients [79,180,181], which was confirmed in humanized mice [132] and differentiated HepaRG cells [130]. In addition, the kinetics of HBV repression in both experimental models correlated with those of IFN activation. Moreover, due to the low infection rate, most of the positive cells were only positive for either HBV or HDV, while the co-infection rate at the single cell level was very low. Thus, HBV repression was likely not a direct effect of HDV markers. Similar to HDV, HCV co-infection also leads to HBV repression. A recent study demonstrated that HBV reactivation after HCV clearance is mainly due to the diminishing HCV-induced IFN response [182]. These studies indicate the important role of HDV-induced IFN response in HBV inhibition.

## 6. Conclusions and Perspectives

In contrast to HBV, HDV replication activates profound IFN-β/λ responses in hepatocytes. MDA5 is the key sensor recognizing HDV replication, but other MDA5-associated factors may also be involved. The IFN response efficiently inhibits the early stages of HDV infection and suppresses HDV RNA amplification during hepatocyte proliferation. However, this response only weakly impairs the intra-nuclear HDV RNA replication in resting cells. HDV may counteract the cellular IFN response through escaping recognition by PRRs and possibly also impairing the IFN signaling pathway. Future work using patient samples and HDV-susceptible infection models with defined genetic modifications of the innate immune signaling pathways will help answer the following questions: (i) What are the statuses of innate immune responses in HDV-infected patients with different viral loads and at different stage of disease progression? (ii) How is HDV RNA recognized, including the viral RNA ligand(s), location of the recognition, roles of the host factors besides MDA5, and the impact of viral factors like HDAg and HBV envelope proteins? (iii) How does the IFN response affect HDV replication/persistence, and how does HDV counteract this affect? These studies will not only help understand the interplay between HDV and the innate immune system but also provide important insights for developing curative therapies against CHD.
